# Ti-Supported Oxide Coatings Based on MWO_4_ (M = Fe, Co, Ni): Plasma Electrolytic Synthesis, Characterization and Catalytic Properties in S, N-Heterocycles Peroxide Oxidation

**DOI:** 10.3390/molecules30091998

**Published:** 2025-04-30

**Authors:** Irina G. Tarkhanova, Vladimir M. Zelikman, Irina V. Lukiyanchuk, Marina S. Vasilyeva, Vladimir V. Tkachev, Vladimir V. Korochentsev, Daria H. Shlyk

**Affiliations:** 1 Department of Chemistry, Lomonosov Moscow State University, Leninskiye Gory, 1/3, Moscow 119991, Russia; 2 Institute of Chemistry, Far Eastern Branch, Russian Academy of Sciences, 100-letya Vladivostoka Prosp., 159, Vladivostok 690022, Russia; lukiyanchuk@ich.dvo.ru (I.V.L.); marina_x@mail.ru (M.S.V.); daria79@list.ru (D.H.S.); 3 Department of Natural Sciences, Far Eastern Federal University, FEFU Campus 10, Ajax Bay, Russky Island, Vladivostok 690922, Russia

**Keywords:** plasma electrolytic oxidation, tungstates, peroxide oxidation, desulfurization, denitrogenation

## Abstract

In this study, catalytically active coatings on titanium were synthesized by plasma electrolytic oxidation (PEO) in aqueous electrolytes based on sodium tungstate with the addition of sodium phosphate or sodium borate and chelate complexes of iron, cobalt or nickel. Taking into account the EDX, XPS and XRD data, the oxide–phosphate coatings (PWFe, PWCo, PWNi) contained crystalline titanium oxide and amorphous tungstates and/or phosphates of iron triad metals. Amorphization was facilitated by high phosphorus concentrations (up to 6 at.%). Replacing phosphate with borate in the electrolyte with Ni(II)-EDTA complexes led to the crystallization of WO_3_ and NiWO_4_ in the PEO coatings (BWNi). All formed PEO coatings were active in reactions of the oxidative desulfurization (ODS) of thiophene and dibenzothiophene and oxidative denitrogenation (ODN) of pyridine, as well as in the simultaneous removal of S- and N-containing substrates from their mixture. The stability of samples with MWO_4_ increased in the following series: PWNi < PWCo < PW < PWFe < BWNi. Replacing phosphate with borate in the electrolyte resulted in the preparation of catalysts with enhanced stability and activity. In contrast to PWM catalysts, the BWNi catalyst had selectivity toward the oxidation of pyridine in its mixture with thiophene.

## 1. Introduction

Plasma electrolytic oxidation (PEO) generates oxide layers on the surface of metals and alloys [1,2,3,4,5]. Under electric spark and microarc discharges, it is possible to form both protective [6,7] and functional coatings [8,9,10]. The PEO technique is promising for the preparation of both secondary carriers [11] and catalytically active oxide layers [12,13] on metal supports. By adjusting the electrical parameters and process duration, this method allows producing porous coatings [14,15] of a certain phase composition with excellent adhesion to the metal support [16]. By combining the electrolyte formula, it makes it possible to apply oxides of one or more elements with catalytic properties to the surface of the metal being processed in “one pot” [17].

The advantage of PEO is not only its one-stage nature, but also the possibility of firmly fixing the catalytically active substance on the surface of the metal or alloy being processed, since an intermediate layer of oxides of the metal or alloy being processed is formed between them [18]. Although the specific surface area of “metal/PEO layer” composites is smaller than that of traditional mineral carriers [17], wide pores, accessibility of the active phase for reacting substrates, and their stability make them promising for liquid-phase catalytic processes involving bulk organic molecules. Among such processes on PEO coatings, the following have been reported: the oxidative dehydrogenation of cyclohexane to cyclohexene [17], biomass gasification [19], photocatalytic degradation of phenol [20] and organic dyes [9,21,22], and oxidation of S- and N-containing substrates of petroleum feedstock [23].

The removal of S-containing compounds from hydrocarbon feedstock by oxidation is an alternative to the hydrotreating method due to its greater safety and simplicity of technological design, since in this case, the reaction can be carried out under mild conditions using eco-friendly hydrogen peroxide or oxygen [24,25,26]. Combinations of oxides of tungsten, vanadium or molybdenum with acidic oxides like P_2_O_5_ make it possible to simulate the corresponding heteropoly acids, which are the most effective catalysts for the peroxide oxidation of S-containing compounds [24,27,28]. Their activity is due to the synergistic action of peroxo complexes of metals of groups V–VI and Brønsted acid centers formed in the presence of hydrogen peroxide. These centers facilitate the coordination of S-containing heterocycles with high nucleophilicity and can cause a distortion of their aromatic structure, which contributes to the oxidation reaction. Over these catalysts, the reaction proceeds by the nucleophilic substitution mechanism. At the same time, it is known that compounds of metals capable of reversible valence change (Fe, Co, Cu, Ce, Cr) can subject the CC bond to oxidation by oxygen or peroxides, as well as by a radical mechanism through the formation of peroxide or superoxide radicals [29]. In addition, many transition metals, such as Ni, are capable of adsorbing the CC bond through coordination interactions [30,31]. According to Refs. [32,33], mixing metal oxides capable of forming isopoly and heteropolyoxoanions and iron triad metal oxides allows combining all the mechanisms of activation of the CC bond, which should increase the efficiency of its oxidation.

The possibility of using W-containing PEO layers on valve metals (Al, Ti) as catalysts for the oxidation of thiophene, one of the most difficult-to-oxidize aromatic compounds of oil, with hydrogen peroxide was first reported in Ref. [34]. Considering the data of Refs. [19,21,22], one can say that PEO layers based on tungsten compounds—oxides and tungstates, including tungstates of transition metals (Mn, Fe, Co, Ni, Cu, Zn)—are promising for the activation of catalytic processes.

Interest in the simultaneous removal of S- and N-containing substrates is due to the tightening of requirements for the content of heteroatomic compounds in petroleum feedstock [35,36,37,38]. As a rule, oxidative denitrogenation (ODN) proceeds more easily than oxidative desulfurization (ODS). On the other hand, the nitrogen content in some petroleum products is not as strictly regulated as that of sulfur (less than 10 ppm). Therefore, in some cases, there is no need to carry out their complete joint removal, which allows reducing the non-target consumption of the oxidizer [39]. On the contrary, some petrochemical processes require the selective removal of N-containing derivatives, which are catalytic poisons. Therefore, the synthesis of compositions that meet all of the above requirements is an urgent task [40].

The purpose of this work is the plasma electrolytic formation of Ti-supported compositions based on P_2_O_5_, WO_3_ and oxides of iron triad metals (Fe, Co, Ni), as well as their use for the oxidation of S- and N-containing heterocycles with hydrogen peroxide. ODS and ODN reactions were carried out both separately and together. In addition, in the catalyst with the least stable oxide layer, phosphorus oxide was replaced by another acidic oxide—B_2_O_3_.

To obtain PEO coatings with B_2_O_3_ instead of P_2_O_5_, sodium phosphate in the phosphate–tungstate electrolyte was replaced by sodium borate. It is known that the electrolyte formula has a significant effect on the PEO process, as well as on the characteristics and properties of the resulting coatings [41]. According to Refs. [42,43], replacing Na_2_HPO_4_ with Na_2_B_4_O_7_ leads to the formation of hierarchical oxide coatings with higher adhesion to the metal, increased roughness and porosity, excellent wettability, and better biological properties. Furthermore, crystalline PEO coatings are formed in borate–tungstate electrolytes with EDTA complexes of transition metals [22], while the presence of phosphates contributes to the production of amorphous coatings. The approach associated with the replacement of the base electrolyte is expected to be effective in improving the activity and stability of PEO catalysts.

The choice of thiophene (T), dibenzothiophene (DBT) and pyridine (Py) as model substrates is due to their presence in light oil fractions and motor fuel. Moreover, T is the most resistant to oxidation among S-heterocycles [24], and Py is a poison for many transition metal catalysts [39]. Therefore, it is important to analyze the possibility of oxidation of S-containing compounds in the presence of N-containing ones.

## 2. Results and Discussion

### 2.1. Preparation and Characterization of Catalysts

The Ti-supported catalysts were synthesized by the PEO technique. The formation curves are given in Figure S1 (Supplementary File). The thickness of the coatings formed in the PW-based electrolytes is about 30 μm. The formation voltage in the BWNi electrolyte is reduced compared to the PWNi electrolyte, which is accompanied by a twofold decrease in the coating thickness (Table 1).

**Figure 1:**
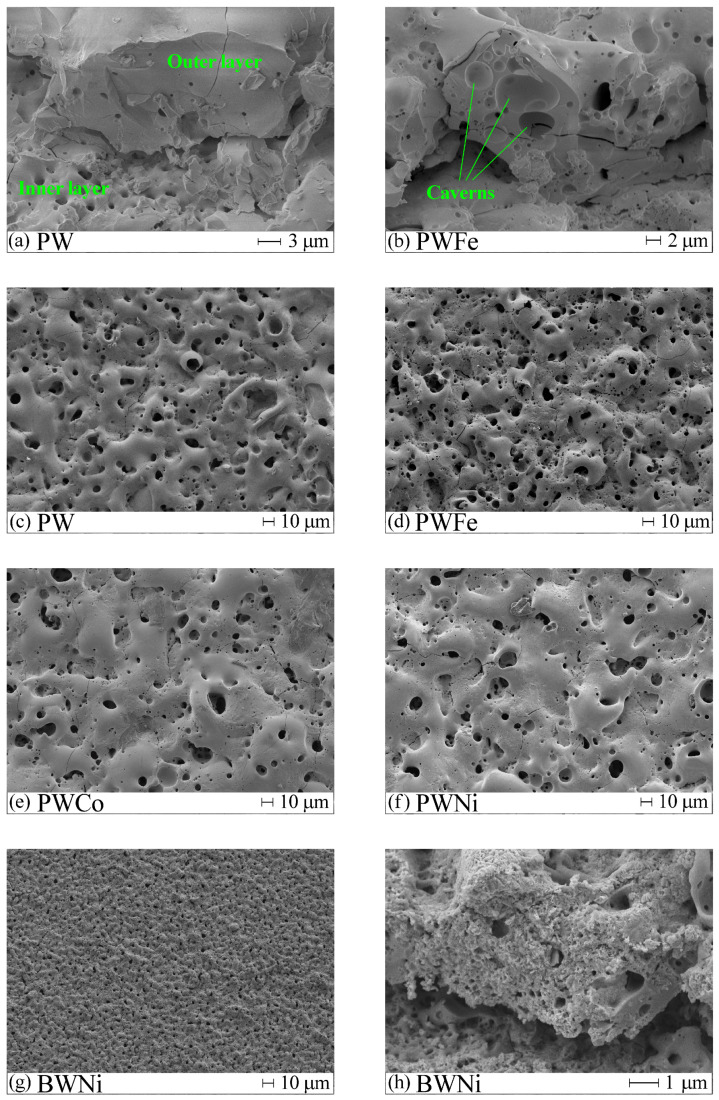
SEM images of the surface of PEO coatings: view at 45° (**a**,**b**,**h**) and top view (**c**–**g**).

The influence of the electrolyte on the surface morphology can be seen in the SEM images in Figure 1. All coatings obtained in phosphate–tungstate electrolytes have a similar surface morphology, with melted areas formed by the rapid cooling of the coating material heated by electrical discharges upon contact with the cold electrolyte, and cracks. The surface is permeated with pores of submicron (up to 1 μm) and micron sizes (1–12 μm). Large pores are obviously caused by powerful electrical discharges, while small ones are traces of gas bubbles, which corresponds to the classification of electrical discharges in Ref. [44].

**Figure 2:**
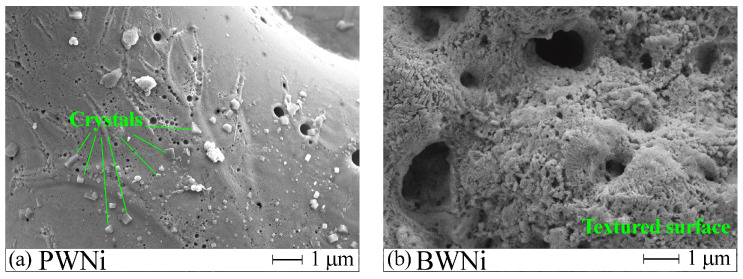
Crystals on the surface of the PWNi sample (**a**) and the textured surface of the BWNi sample (**b**).

Replacing the PWNi electrolyte with BWNi results in the disappearance of large pores and cavities (Figure 1g,h). Comparing the surface morphology of the PWNi and BWNi samples in high-magnification images (Figure 2), one can see the difference in the surface formations. These are regularly faceted crystals on the surface of the PWNi sample (Figure 2a) and a completely textured surface in the case of the BWNi sample (Figure 2b).

It should be noted that all the studied coatings have a two-layer structure with a dense fine-pored inner layer adjacent to titanium and a loose outer layer. Examples are shown in Figure 1a,b,h. According to EDX data, the inner layers contain more titanium, while the outer layers are enriched with electrolyte elements (Table 2).

The introduction of EDTA complexes of transition metals into the base PW electrolyte did not reduce the concentration of tungsten in the formed coatings. Comparing the elemental compositions of the PEO coatings obtained in PWNi and BWNi electrolytes, it can be noted that the latter contain less titanium, more tungsten and nickel, and boron instead of phosphorus.

**Figure 3:**
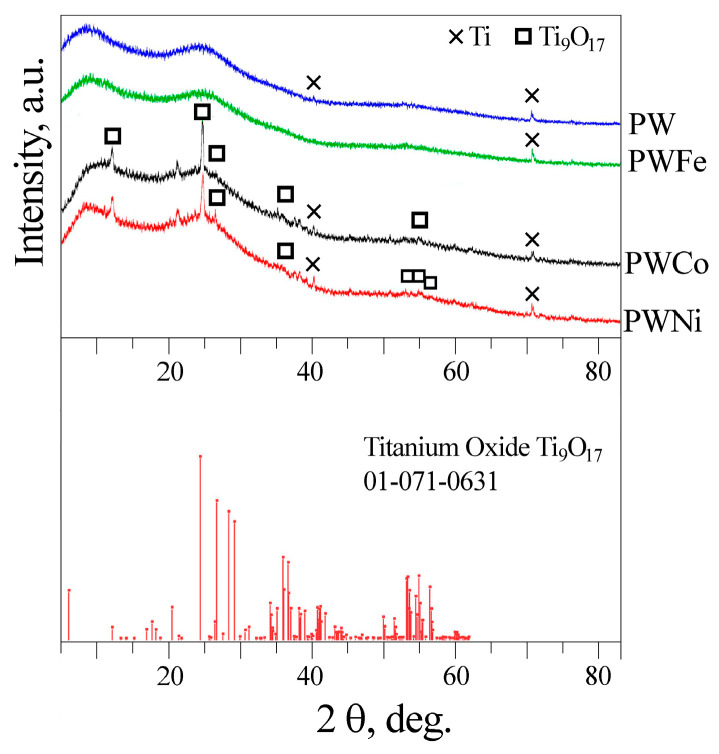
XRD patterns of PEO-coated samples PW and PWM, where M = Fe, Co, Ni.

The XRD patterns of the PW and PWM (M = Fe, Co, Ni) samples are shown in Figure 3. In all cases, peaks of titanium from the substrate are visible. The XRD patterns of the PWNi and PWCo samples contain peaks that can be attributed to partially reduced titanium dioxide Ti_9_O_17_. Since TiO_2_ is always formed at the initial stage of PEO treatment of titanium [18], it should be present in the other two samples. In addition, all XRD patterns show two amorphous halos in the regions of 2θ = 5–17° and 20–32°. At the same time, crystalline compounds of the tungsten and iron triad metals were not detected. In Ref. [45], the broad peak near 2θ = 20–35° observed in the samples processed at low current densities in an electrolyte containing 0.01 mol/L K_4_P_2_O_7_ and 0.02 mol/L KOH was explained by the formation of an amorphous P-containing phase. Considering the high concentrations of phosphorus in the coatings and the possibility of glass formation in the WO_3_-P_2_O_5_ [46] and WO_3_-TiO_2_-P_2_O_5_ [47] systems, the presence of amorphous phosphates and tungstates of iron triad metals in our samples can be assumed.

**Figure 4:**
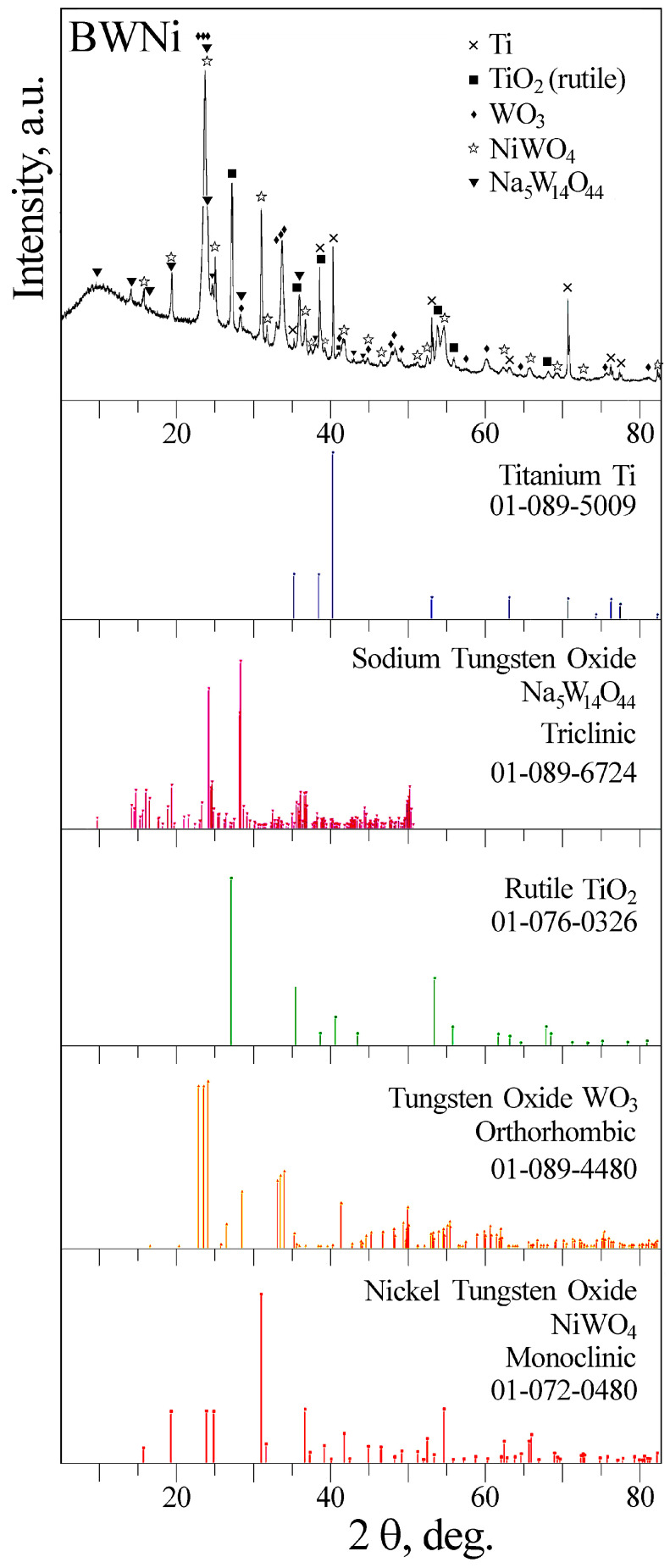
XRD pattern of BWNi sample.

Replacing phosphate with borate in the base electrolyte (BWNi) resulted in crystallized coatings containing rutile, tungsten trioxide, nickel tungstate, and sodium tungsten bronze-like compound (Figure 4, Figures S3 and S4). The fact that borate in the BW electrolyte promotes the crystallization of tungsten compounds in PEO coatings was previously reported in Ref. [22].

**Figure 5:**
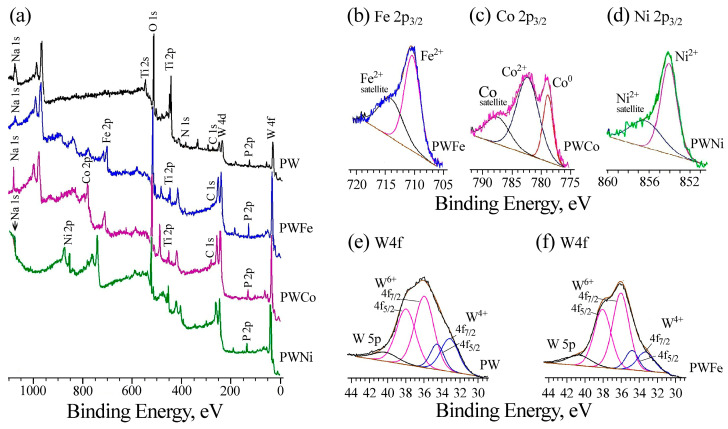
Survey (**a**) XPS spectra and regional spectra of Fe 2p_3/2_ (**b**), Co 2p_3/2_ (**c**), Ni 2p_3/2_ (**d**), and W 4f (**e**,**f**) electrons for subsurface layers of PW (**a**,**e**), PWFe (**a**,**b**,**f**), PWCo (**a**,**c**), and PWNi (**a**,**d**) samples.

The chemical composition of the subsurface layers of the samples was determined based on the XPS data (Figure 5, Table 3). Since the surface layers are heavily contaminated with carbon (18–41 at. % C, Table S1 in Supplementary Materials), the samples were bombarded with argon ions, which resulted in a decrease in the carbon concentration (to 2.5–7.2 at.% for PWM samples and to 19.4 at.% for PWNi) and etching of the surface layers of the PEO coatings to a depth of ~30 Å.

The C1s band is contributed by oxidized (*E*_b_~288 eV), aliphatic (C=C, C-H, *E*_b_ = 285 eV) and carbide carbon (*E*_b_~283 eV). The formation of carbide carbon in PEO coatings on titanium after etching was noted in [48]. The O1s bands are contributed by the P-O and C-O structures with binding energies in the range of 532–533 eV and the M-O state (*E*_b_ = 530.6–531.5 eV). Titanium is predominantly in the Ti^4+^ state. In the region of lower binding energies, a contribution from the Ti-N state is observed, which is confirmed by the presence of the N 1s peak with a binding energy in the range of 400 eV. Phosphorus is in the P^5+^ state, which corresponds to our assumptions about the presence of phosphates or phosphate glasses in the coatings. All coatings are characterized by a large contribution of tungsten, which is in the W^6+^ and W^4+^ states (Table 3), while only W^6+^ is present in the surface layers (Table S1). When iron triad metals are introduced into the coatings, the proportion of W^6+^ increases slightly, and the W 4f band itself becomes narrower (Figure 5e,f). The oxidation state of 3d metals is traditional for such systems. Iron and nickel are in the oxidation state +2, although Fe^3+^ is also present in the surface layers of the PWFe sample (Table S1). In the Co 2p spectrum after etching, both Co^2+^ and Co^0^ are observed with *E*_b_ of 782.0 and 778.5 eV, respectively.

**Figure 6:**
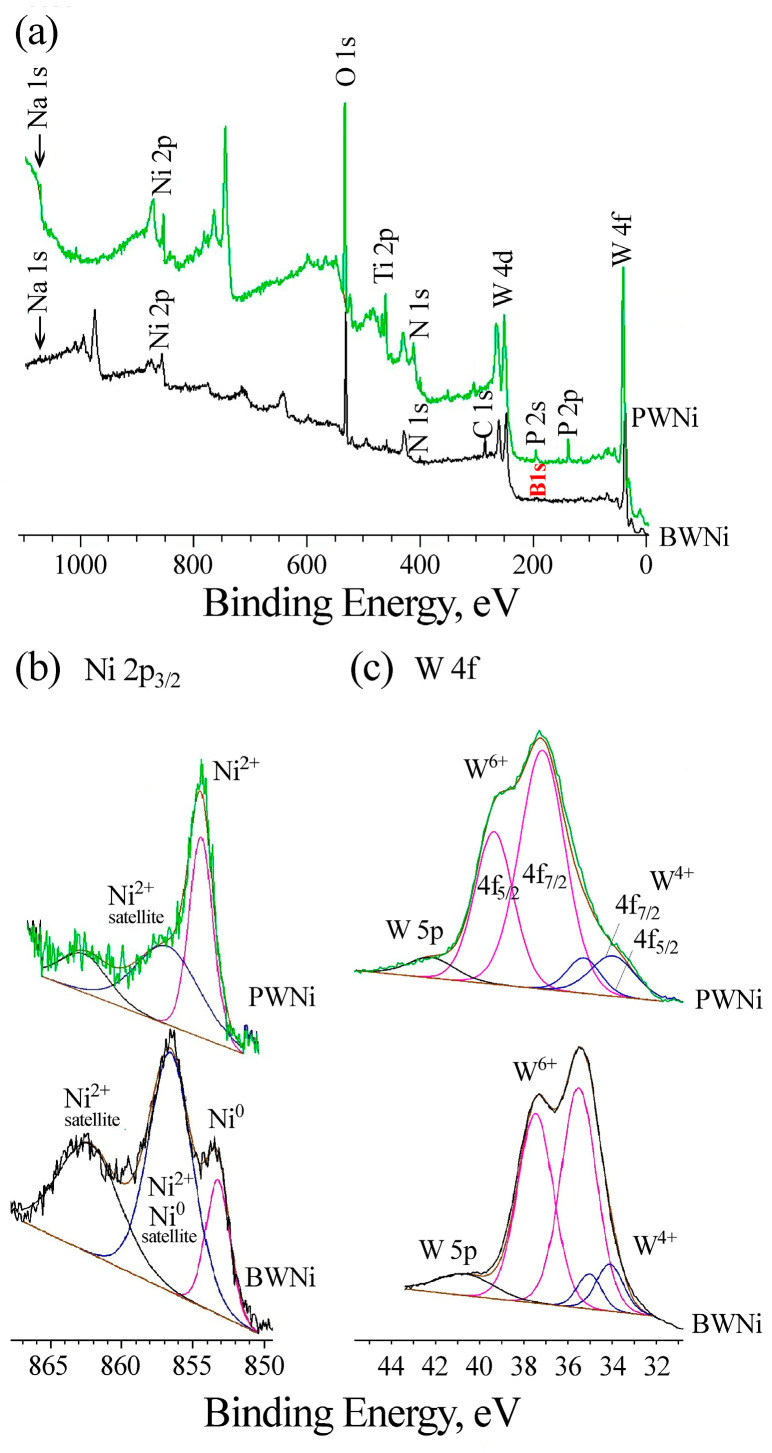
Comparison of XPS spectra of PWNi and BWNi samples: (**a**) survey spectra, (**b**) Ni 2p_3/2_ spectra, (**c**) W4f spectra.

Comparison of the XPS spectra of PWNi and BWNi samples (Figure 6) shows that when phosphate is replaced by borate in the electrolyte, B^3+^ is incorporated into the coating composition instead of P^5+^, the W4f spectrum narrows, and Ni^0^ appears along with Ni^2+^ after etching the samples. A decrease in the width of the W4f spectrum is accompanied by the crystallization of nickel tungstates in the coating composition, according to XRD data (Figure 4).

A decrease in the oxidation degree of metals during their incorporation from the electrolyte into the PEO coatings was also noted in Refs. [49,50]. The reason for the appearance of reduced (Ni^0^, Co^0^) or partially reduced metals (W^4+^) in the composition of anodic coatings may be the action of hydrogen released at the anode during the thermolysis of water molecules [51], thermal decomposition of M−EDTA complexes [52], and also bombardment of the surface with high-energy argon ions [53].

### 2.2. Catalytic Properties

The dosage of oxidant was marked as O/S (the molar ratio of H_2_O_2_ to sulfur). When the O/S molar ratio reached up to 4 or 8, the desulfurization rate significantly increased; this was because of the increasing production quantity of catalytic activity species [4]. However, the sulfur removal slightly decreased when the O/S molar ratio was increased to 12. The catalyst deactivation could be attributed to the occupation of catalyst surface by excessive water in the ODS process [19]. Taking the desulfurization efficiency into account, an O/S molar ratio of 4 was selected as the optimal conditions.

The conditions for the catalysis were chosen based on the analysis of our previous studies and literature data [54,55]. A temperature of 60 °C is optimal since it ensures a sufficiently high rate of the target oxidation reaction, while its increase leads to an acceleration of the side reaction of peroxide decomposition and the destruction of metal peroxo complexes responsible for catalysis [56]. According to Ref. [57], the optimal peroxide to substrate ratio ranges from 4:1 to 8:1, depending on the reactivity of the substrate and the nature of the reaction products. It is known that the oxidation of thiophene can proceed up to the formation of SO_4_^2−^ and CO_2_, with thiophene being one of the most difficult-to-oxidize heterocycles [34,58]. Therefore, in the case of our reaction involving thiophene, the smallest amount of peroxide was used in order to prevent deep oxidation and reduce the contribution of the side reaction of peroxide decomposition.

**Figure 7:**
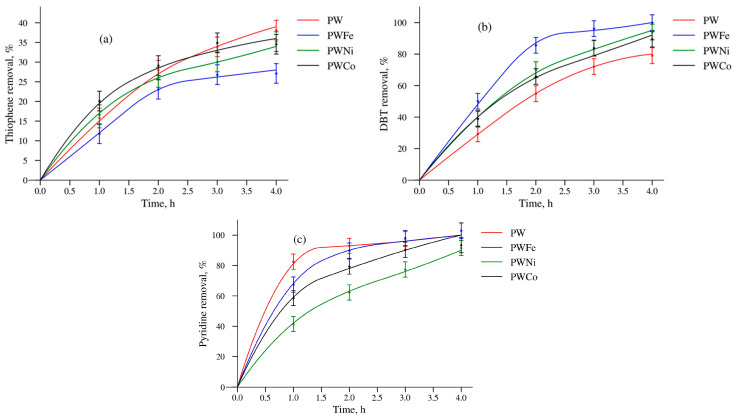
Removal of thiophene (**a**), DBT (**b**) and pyridine (**c**) on PW, PWFe, PWNi and PWCo catalysts in oxidation reactions.

The catalytic properties of the PW and PWM samples in the oxidation of T, DBT and Py with hydrogen peroxide are presented in Figure 7. The catalytic properties of the BWNi sample will be presented later.

It is evident that the activity of the samples depends on the nature of the substrate. In the reaction with thiophene, the PWCo sample was the most active at the initial stage, but after 4 h, the PW sample showed the best results. The PWFe sample turned out to be the most stable: a high degree of thiophene removal was maintained for five cycles. Such regularities can be explained by the greater stability of the PWFe oxide layer on the surface of the titanium support leading to minimal weight loss (Table 4).

As demonstrated by the analysis of the data in Table 4, the activity of Ni- or Co-containing catalysts in the oxidation of all substrates can be associated with the leaching of the active phase components into the solution and the appearance of soluble catalytic complexes. On the other hand, in the reaction with DBT and pyridine, the PWFe catalyst was the most active both at the initial stage and at the end of the reaction, although its weight loss was minimal (Table 4). Thus, the leaching of the active substance into the solution is not a necessary condition for high activity from the PEO catalyst.

From the analysis of the data in Table 4, it follows that the stability of samples with amorphous MWO_4_ increases in the following series: PWNi < PWCo < PW < PWFe, with the PWNi catalyst being the least stable. At the same time, in the absence of phosphorus oxide, mixed oxide layers based on WO_3_ + NiWO_4_ were quite effective in the ODS and ODN reactions [59]. To increase the stability of the Ni-, W-containing PEO catalyst, Na_3_PO_4_ was replaced by Na_2_B_4_O_7_ in the electrolyte. Indeed, in this case, we obtained a BWNi sample that was active in the reactions of oxidation of T, DBT and Py and more stable compared to the PWNi sample (Table 4 and Table 5).

The oxidation of thiophene on heterogeneous catalysts can be described using the first-order kinetic equation (ln *C*_0_/*C* = k*t*) [60]. Table 5 shows the rate constants of substrate oxidation under optimal conditions for the most stable samples.

Taking into account that thiophene is converted to SO_4_^2−^ [34], the highest H_2_O_2_ efficiency (more than 45%) was observed for its oxidation on PW and BWNi catalysts. The BWNi sample exhibited greater oxidation activity with thiophene and dibenzothiophene with the least leaching.

**Figure 8:**
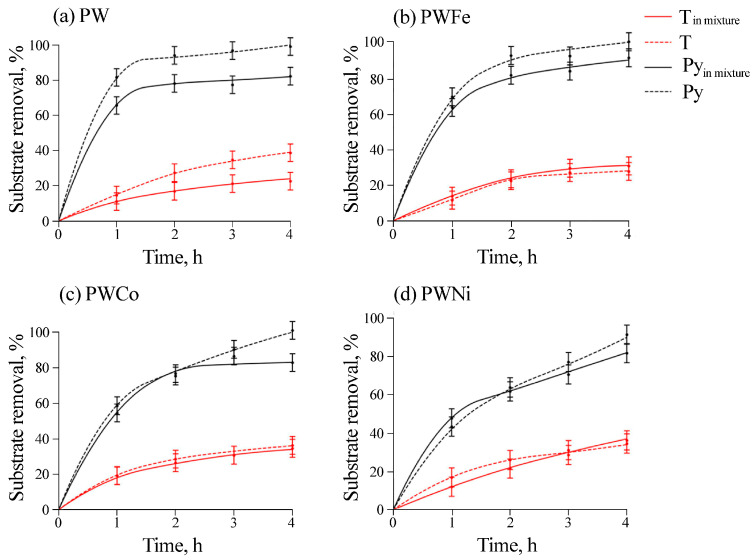
Separate and simultaneous oxidation of thiophene and pyridine oxidation by hydrogen peroxide on (**a**) PW, (**b**) PWFe, (**c**) PWCo and (**d**) PWNi catalysts.

All the catalysts were tested in co-oxidation reactions of thiophene and pyridine (Figure 8). As demonstrated by the analysis in Figure 8, the presence of pyridine affects the oxidation of thiophene only over the PW catalyst. Seemingly, in this case, the oxidation of pyridine occurs mainly on the surface of the composite (see Table 4), and the products of its transformation block the active centers and inhibit the oxidation of thiophene.

To confirm this assumption, the surface of the PW catalyst after the reaction with thiophene and its mixture with pyridine was analyzed by XPS. The data are presented in Table 6 and Figures S5 and S6.

The analysis in Table 6 shows that after the reaction with T, oxygen derivatives of sulfur (~0.8 at.% S as SO_4_^2−^) were present on the surface of the PW catalyst. In addition, the surface contained the products of the transformation of nitrogen compounds of the catalyst: 0.3 at.% N in the form of oxidation derivatives (O-NR_3_) and 3.0 at.% N in the form of quaternary ammonium derivatives arising due to the interaction with sulfuric acid. After the reaction with the mixture of substrates (T + Py), the concentrations of NR_4_^+^ and oxygen derivatives of sulfur decreased to 1.5 at.% and 0.2 at.%, respectively, but the amount of oxygen derivatives of nitrogen increased to 1.8 at.%. Thus, it can be concluded that the surface was covered mainly with nitrogen derivatives, which hindered the reaction of thiophene.

On the contrary, an insignificant influence of thiophene on pyridine oxidation was observed for another leaching-resistant catalyst, PWFe, as well as for PWCo. When using the most unstable catalyst, PWNi, a complete absence of the influence of both substrates was observed: seemingly, the release of ions into the solution and the formation of homogeneous active complexes allow the oxidation reactions of thiophene and pyridine to proceed independently of each other.

**Figure 9:**
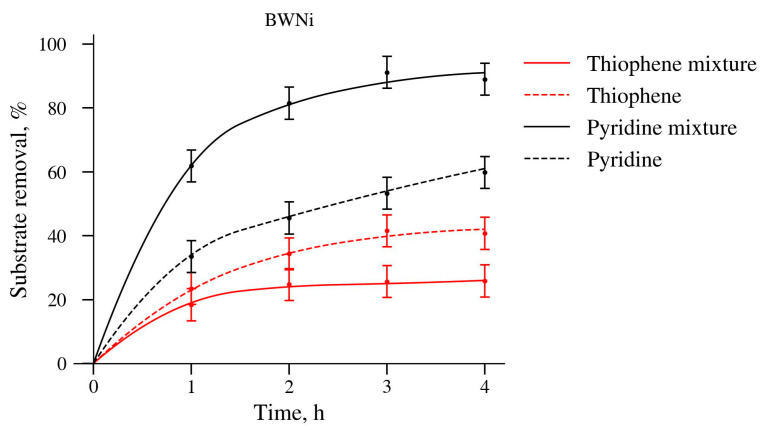
Separate and simultaneous oxidation of thiophene and pyridine by hydrogen peroxide on BWNi catalyst.

Compared with the PWNi sample, the BWNi sample containing crystalline nickel and tungsten compounds was quite active in oxidation reactions and was more stable (Table 4 and Table 5). Analysis of Figure 9 confirms the earlier conclusion that, for a stable catalyst, the mutual influence of substrates in their simultaneous oxidation is very significant and is seemingly related to the blocking of active centers on the surface.

As with the PW catalyst, the decrease in thiophene conversion in the mixture on BWNi can be explained by the strong binding of pyridine or the products of its oxidation with the sample surface. The nature of such a strong acceleration of the pyridine reaction in the presence of thiophene can be related to the appearance of sulfonic acid that binds basic pyridine and exhibits activity in catalysis.

Thus, the formation of crystalline coatings containing NiWO_4_ and WO_3_ and the appearance of metallic nickel (Ni^0^) in the BWNi subsurface layers increased the stability of the catalyst (Table 4) and led to a selectivity for pyridine oxidation in the presence of thiophene (Figure 9).

## 3. Materials and Methods

### 3.1. Pre-Treatment of Titanium Samples and Preparation of Catalysts

The supports for applying catalytic coatings were made of sheet titanium grade VT1–0 (wt. %: 0.7 Al, 0.25 Fe, 0.10 Si, 0.07 C, 0.04 N, 0.2 O, 0.01 H; other impurities—0.30 and Ti—the rest). The dimensions of the plates were 2.0 × 2.0 × 0.05 mm. The titanium plates were subjected to mechanical grinding, chemical polishing in a mixture of concentrated acids (HF:HNO_3_ = 1:3 in volume ratio) at 60−80 °C, washing with distilled water, and drying in air at 70 °C.

To apply catalytically active compositions to the surface of the titanium samples, anodic treatment in the spark and microarc electric discharge mode was used. The PEO treatment was carried out in an electrochemical cell—a 1 L polypropylene beaker with the electrolyte, into which the titanium sample being treated and a counter electrode were placed. The sample was connected to the positive pole of the current source—a PC-controlled TER4–100/460N–2–2UKhL4 thyristor unit (Russia) operating in the unipolar mode. A hollow nickel coil served as a counter electrode. Tap water was passed through this coil to cool the electrolyte, which was magnetically stirred so that the temperature did not exceed 35 °C during the PEO process. After PEO treatment, the samples were washed with distilled water and dried in air.

The parameters of PEO treatment and electrolyte formulas are given in Table 1. The electrolytes were prepared using distilled water and commercially available reagent-grade chemicals: Na_3_PO_4_·12H_2_O, Na_2_B_4_O_7_·10H_2_O, Na_2_WO_4_·2H_2_O, (NH_4_)_2_Fe(SO_4_)_2_·6H_2_O, Co(CH_3_COO)_2_·4H_2_O, Ni(CH_3_COO)_2_·4H_2_O, and C_10_H_14_N_2_Na_2_·2H_2_O. A solution of 0.05 M Na_3_PO_4_ + 0.1 M Na_2_WO_4_ (in a special case, 0.05 M Na_2_B_4_O_7_ + 0.1 M Na_2_WO_4_) was used as the base electrolyte, into which EDTA complexes of iron triad metals were introduced. Acetates of cobalt and nickel were used as sources of Co(II) and Ni(II), and Mohr’s salt served as a source of Fe(II).

### 3.2. Characterization of Catalyst Surfaces

The Ti-supported catalysts were characterized by X-ray diffraction (XRD), energy-dispersive X-ray (EDX) analysis, scanning electron microscopy (SEM), and X-ray photoelectron spectroscopy (XPS).

X-ray diffraction patterns of PEO-coated samples were recorded on a STADI P diffractometer (STOE & Cie GmbH, Darmstadt, Germany) in the range of 2θ angles from 5 to 80° with a step of 0.5° (Ni filter). Phase identification was performed based on the PDF-2 database.

The morphology and elemental composition of sample surfaces were investigated using high-resolution scanning electron microscopy (SEM) and energy-dispersive X-ray (EDX) microanalysis. Experiments were carried out using a ZEISS ULTRA 55+ scanning electron microscope (Carl Zeiss AG, Oberkochen, Germany) equipped with an Oxford X-MAX EDX detector (Oxford Instruments PLC, Abingdon, Oxfordshire, UK). Images were acquired at an accelerating voltage of 5 kV, and the edges were observed at a 45-degree angle. Elemental analysis was performed at an accelerating voltage of 20 kV. The scanning beam penetration depth was approximately 1 µm, and the sizes of the analyzed areas for determining the overall composition of the coatings were at least 50 × 50 µm. Preliminary intensity calibration was performed using a cobalt standard.

The X-ray photoelectron spectra were recorded on a Specs ultra-high vacuum photoelectron spectrometer (SPECS GmbH—Surface Analysis and Computer Technology, Berlin, Germany) with a hemispherical electrostatic analyzer (radius of curvature—150 mm) and Al*K*_α_ radiation source (source energy—1456.6 eV) at pressures in the analysis chamber of 10^−6^–10^−7^ Pa. Survey spectra were recorded in the range of 1100−0 eV. The spectra were processed using the CasaXPS Version 2.3.12 program. The calibration of the electron binding energy scale was performed using the internal standard technique for which C1s level (285.0 eV) was chosen. The spread function of the spectrometer in the mode of characteristic atomic level registration, which was determined by the shape of the Ag 3d_5/2_ band, had a half-width of 1.2 eV. To remove carbon contamination, each sample was bombarded with high-energy Ar^+^ ions, etching the surface to a depth of 30 Å. The composition of the subsurface layers was then analyzed.

The thickness of the formed PEO coatings was measured 10–12 times on both sides of each sample using a VT-201 eddy current thickness gauge (Control, Measurement, Diagnostics LLC, Khimki, Russia). The obtained data were averaged.

### 3.3. Catalytic Testing and Reaction Product Analysis

For catalytic testing, the PEO-coated titanium samples were cut into 0.1 g pieces. These plates and 5 mL of model solution (1 wt. % thiophene, dibenzothiophene, or pyridine in isooctane) were placed into a jacketed glass reactor. To carry out the reaction with T and Py, the corresponding solutions (2.5 mL + 2.5 mL) were simultaneously poured into the reactor. The reaction mixture was magnetically stirred then heated to 60 °C, after which 0.2 mL of 30% H_2_O_2_ solutions was added. After the completion of the reaction, the liquid phase was decanted, and the catalyst was washed with isooctane. Next, a new portion of the reagents was placed in the reactor, and the tests were carried out in the same way for 5 cycles.

Samples of the organic phase were taken from the reaction mixture every hour and analyzed by gas–liquid chromatography on a Crystal 4000 instrument (Meta-Chrome LLC, Yoshkar-Ola, Russia, the measurement error was ±3.0%) equipped with a 30 m long Zebron ZB-1 capillary column (Phenomenex Inc., Torrance, CA, USA, 100% dimethylpolysiloxane as the liquid phase) and a flame ionization detector (FID) using an internal standard (n-nonane or n-dodecane).

## 4. Conclusions

1. Ti-supported catalysts with amorphous tungstates of iron triad metals were successfully synthesized by the PEO technique in phosphate–tungstate electrolytes with chelate complexes of iron, cobalt or nickel. The obtained catalysts showed activity in both separate and combined oxidation of S- and N-containing organic substrates (thiophene, dibenzothiophene, pyridine).

2. Replacing the phosphate–tungstate electrolyte with a borate–tungstate electrolyte resulted in the production of crystalline PEO coatings containing NiWO_4_ and WO_3_, the appearance of metallic nickel (Ni^0^) in the subsurface layers, an increase in the stability of the catalyst oxide layer, and the appearance of selectivity towards pyridine in the simultaneous oxidation of thiophene and pyridine. The resulting BWNi sample exhibited greater oxidation activity with thiophene (H_2_O_2_ efficiency more than 45%) and dibenzothiophene with the least leaching.

## Figures and Tables

**Table 1:** Sample and electrolyte designations and coating thicknesses, electrolyte formulas, and electric modes.

Label	Base Solution	Additives	*i* [A/cm^2^]	*t* [min]	U_max_ [V]	U_f_ [V]	*h* [μm]
PW	0.05 M Na_3_PO_4_ + 0.1 M Na_2_WO_4_	No	0.1	10	106 ± 2	94 ± 2	29 ± 3
PWFe	0.05 M Na_3_PO_4_ + 0.1 M Na_2_WO_4_	0.05 M Na_2_H_2_EDTA + 0.05 M Fe(NH_4_)_2_(SO_4_)_2_	0.05	10	98 ± 1	92 ± 2	36 ± 3
PWCo	0.05 M Na_3_PO_4_ + 0.1 M Na_2_WO_4_	0.05 M Na_2_H_2_EDTA + 0.05 M Co(CH_3_COO)_2_	0.1	10	145 ± 3	141 ± 5	35 ± 4
PWNi	0.05 M Na_3_PO_4_ + 0.1 M Na_2_WO_4_	0.05 M Na_2_H_2_EDTA + 0.05 M Ni(CH_3_COO)_2_	0.1	10	150 ± 2	144 ± 3	32 ±3
BWNi	0.05 M Na_2_B_4_O_7_ + 0.1 M Na_2_WO_4_	0.05 M Na_2_H_2_EDTA + 0.05 M Ni(CH_3_COO)_2_	0.1	10	90 ± 2	89 ± 2	17 ± 3

Notes: U_max_—maximum voltage achieved during PEO process, U_f_—final voltage of coating formation.

**Table 2:** Elemental composition of outer and inner layers of PEO coatings according to EDX data.

Catalyst	Layer	Elemental Composition [at. %]							
		C	O	Na	P	B	Ti	W	M
PW	Outer	8.2	69.2	2.2	5.3	-	9.7	5.4	-
	Inner	3.4	68.2	0.1	1.5	-	25.1	1.7	-
PWFe	Outer	7.9	68.8	1.9	6.0	-	6.1	5.5	3.8 Fe
	Inner	4.3	67.8	0.2	2.3	-	22.1	2.5	0.8 Fe
PWCo	Outer	7.6	66.2	3.2	4.8	-	5.6	8.1	4.5 Co
	Inner	3.1	68.9	0.2	2.1	-	20.2	4.7	0.8 Co
PWNi	Outer	7.4	66.4	3.3	4.9	-	5.4	8.5	4.1 Ni
	Inner	3.1	68.7	0.7	2.4	-	19.2	5.0	0.9 Ni
BWNi	Outer	12.3	59.4	0.5	-	8.1	1.9	10.6	7.3 Ni
	Inner	3.8	66.3	0.3	-	0.0	26.2	3.1	0.3 Ni

Notes: M = Fe, Co or Ni; “-” element is absent.

**Table 3:** Binding energies *E*_b_ [eV] and concentrations of elements *C* [at. %] in the subsurface layers of the samples according to the XPS data.

Line	PW		PWFe		PWCo		PWNi		BWNi		Group
	*C*	*E*_b_	*C*	*E*_b_	*C*	*E*_b_	*C*	*E*_b_	*C*	*E*_b_	
Na1s	3.6	1071.8	0.7	1072.0	4.6	1072.3	1.2	1072.0	0.6	1072.0	Na^+^
Fe 2p_3/2_			6.6	710.4							Fe^2+^
Co 2p_3/2_					7.6	782.0					Co^2+^
					1.9	778.5					Co^0^
Ni 2p_3/2_							3.2	854.2	6.6	856.5	Ni^2+^
									2.0	853.2	Ni^0^
O 1s	14.6	532.1	9.6	532.7	15.2	532.6	9.7	532.0	12.7	531.9	PO_x_, CO_x_, BO_x_
	46.3	530.6	48.0	531.4	37.2	531.5	50.7	530.8	36.3	530.9	MO_x_
Ti 2p_3/2_	13.2	459.0	3.6	459.5	3.4	459.2	4.2	458.9	1.7	459.2	Ti^4+^
	2.1	457.8	1.2	458.1	0.5	457.9	1.1	457.7	0.3	458.1	Ti−N
N 1s	0.9	399.8	1.2	400.2	1.0	399.7	0.7	399.5	2.9	400.0	N−Ti
	2.3	397.2	3.0	397.7	1.2	397.4	3.0	397.4	0.9	397.0	N−N, N−H
C 1s	1.3	288.5	0.8	287.1	1.7	287.0			3.6	289.0	O=C(H)−O, COC
	4.7	285.0	1.1	285.0	5.1	284.9	2.6	285.0	15.0	285.0	CC, CH
	1.2	281.5	0.6	283.6	1.0	283.6			0.8	283.4	CW, CTi
P 2p	5.9	133.6	10.53	134.0	6.7	133.5	6.7	134.8			P^5+^ (P_2_O_5_)
B 1s									3.5	191.0	B^3+^ (B_2_O_3_)
W 4f_7/2_	2.7	36.1	10.6	36.1	9.0	35.7	13.3	36.1	11.0	35.4	W^6+^
	1.2	33.1	2.7	33.4	3.9	33.3	3.5	33.4	2.0	34.0	W^4+^
Total	100		100.2		100.0		99.9		99.9		

**Table 4:** Weight loss (%) of samples after five oxidation cycles.

Catalysts	Thiophene	DBT	Pyridine
PW	6	5	4
PWFe	1.5	2	4
PWCo	11	13	5
PWNi	16	14	14
BWNi	2	4	2

**Table 5:** Rate constants [×10^3^ 1/c] of substrate oxidation under optimal conditions.

Catalysts	Thiophene	DBT	Pyridine
PW	0.045	0.073	0.34
PWFe	0.036	0.18	0.36
BWNi	0.048	0.2	0.13

**Table 6:** Binding energies *E*_b_ [eV] and concentrations of elements *C* [at. %] in the surface layers of PW after its interaction with thiophene and its mixture with pyridine according to the XPS data.

Line	After Interaction with Thiophene		After Interaction with Thiophene + Pyridine Mixture		Group
	*C*	*E*_b_	*C*	*E*_b_	
O 1s	4.4	532.8	4.0	533.0	C-O
	15.8	531.7	20.9	531.6	PO_x_, CO_x_, BO_x_
	29.4	530.5	23.2	530.4	O^2−^ (M-O)
Ti 2p_3/2_	4.4	458.8	6.7	458.8	Ti^4+^
N 1s	0.3	402.8	1.8	402.8	O-NR_3_
	3.0	401.6	1.5	401.5	NR_4_^+^
	0.9	399.7	1.2	399.8	N-Ti
	2.3	288.3	3.9	288.5	O-C=O
C 1s	7.9	286.0	9.9	286.2	C-O, C-N
	23.9	284.4	21.6	284.7	C-C
S 2p_3/2_	0.8	167.7	0.2	167.7	SO_4_^2−^
P 2p	2.5	133.3	2.7	133.3	P^5+^
W 4f_7/2_	4.4	35.5	2.4	35.5	W^6+^

## Data Availability

Data will be made available on request.

## Supplementary Materials

The following supporting information can be downloaded at: https://www.mdpi.com/article/10.3390/molecules30091998/s1, Figure S1: Voltage–time responses during PEO treatment of titanium samples in PW, PWM (M = Fe, Co, Ni) and BWNi electrolytes. The formation curves were obtained by averaging 3–5 scans in each electrolyte. Figure S2: An example of determining the elemental composition of BWNi sample on the outer and inner surface areas. Spectrum 1 refers to the outer layer, while spectra 2 and 3 characterize the delamination areas (the surface of the inner layer). Figure S3: XRD pattern of PWNi sample. Figure S4: XRD pattern of BWNi sample. Figure S5: Survey and regional S2p XPS spectra of sample PW after the thiophene oxidation reaction. Figure S6: Survey and regional N1s XPS spectra of sample PW after the oxidation of the thiophene—pyridine mixture. Table S1: Binding energies *E*_b_ and element concentrations *C* for surface and subsurface layers of PEO composites.

## Abbreviations

The following abbreviations are used in this manuscript:

PEO	plasma electrolytic oxidation
ODS	oxidative desulfurization
ODN	oxidative denitrogenation
EDX	energy-dispersive X-ray spectroscopy
XPS	X-ray photoelectron spectroscopy
XRD	X-ray diffraction analysis
