# Supplementary material for: Ti-Supported Oxide Coatings Based on MWO4 (M = Fe, Co, Ni): Plasma Electrolytic Synthesis, Characterization and Catalytic Properties in S, N-Heterocycles Peroxide Oxidation
Source: Molecules. 2025 Apr 30;30(9):1998. doi: 10.3390/molecules30091998 (PMC12073619; doi:10.3390/molecules30091998)
Supplement: Supplementary file 1 [file molecules-30-01998-s001.zip › molecules-3577586-supplementary.pdf]

Supplementary Material for on-line publication only

Ti-supported oxide-phosphate coatings based on MWO_4 (M=Fe, Co, Ni): plasma electrolytic synthesis, characterization and catalytic properties in S, N-heterocycles peroxide oxidation

Irina G. Tarkhanova [1,*], Vladimir M. Zelikman [1], Irina V. Lukiyanchuk [2], Marina S. Vasilyeva [2,3], Vladimir V. Tkachev [4], Vladimir V. Korochentsev [2] and Daria H. Shlyk [2]

[1] Department of Chemistry, Lomonosov Moscow State University, Leninskiye Gory, 1/3, Moscow 119991, Russia
[2] Institute of Chemistry, Far Eastern Branch, Russian Academy of Sciences, 100-letya Vladivostoka Prosp., 159, Vladivostok 690022, Russia
[3] Institute of High Technologies and Advanced Materials, Department of Chemistry and Materials, Far Eastern Federal University, FEFU Campus 10, Ajax Bay, Russky Island, Vladivostok 690922, Russia
[4] Faculty of Material Science, MSU-BIT University, 1 International University Park Road, Dayun New Town, Shenzhen 518172, China
* Correspondence: itar_msu@mail.ru

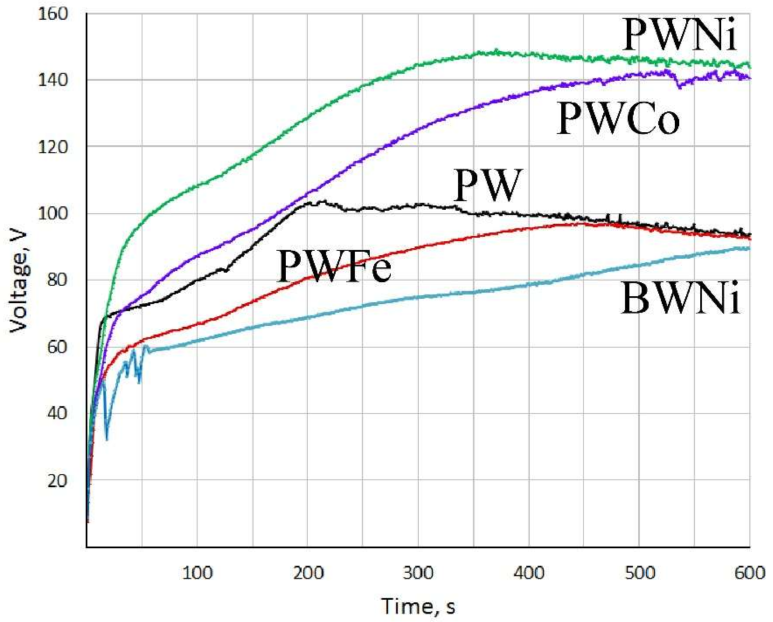

Figure S1. Voltage-time responses during PEO treatment of titanium samples in PW, PWM (M=Fe, Co, Ni) and BWNi electrolytes. The formation curves were obtained by averaging 3-5 scans in each electrolyte. The electrolyte formulas and formation modes are given in Table 1.

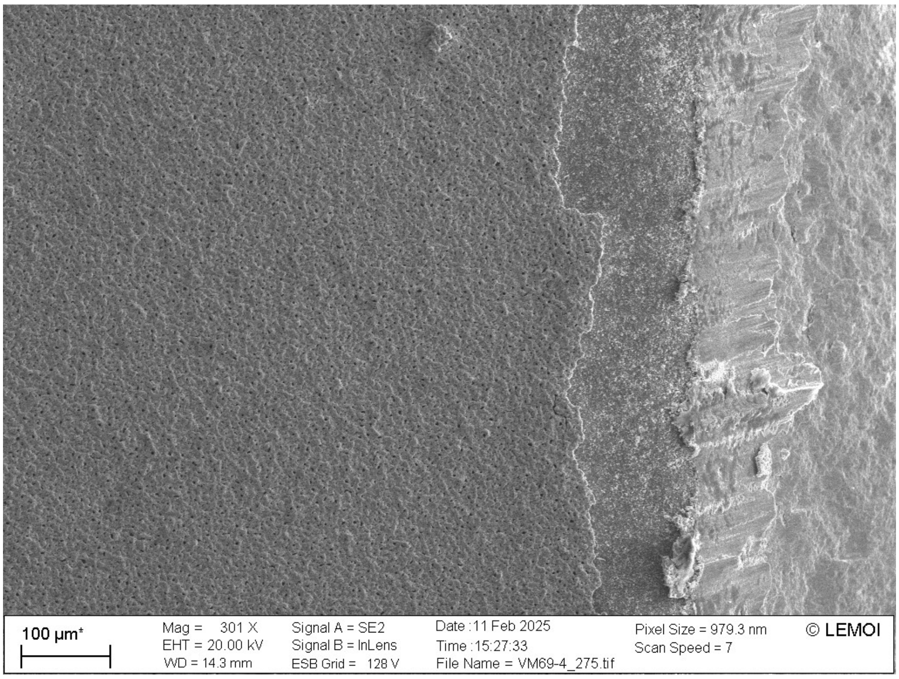

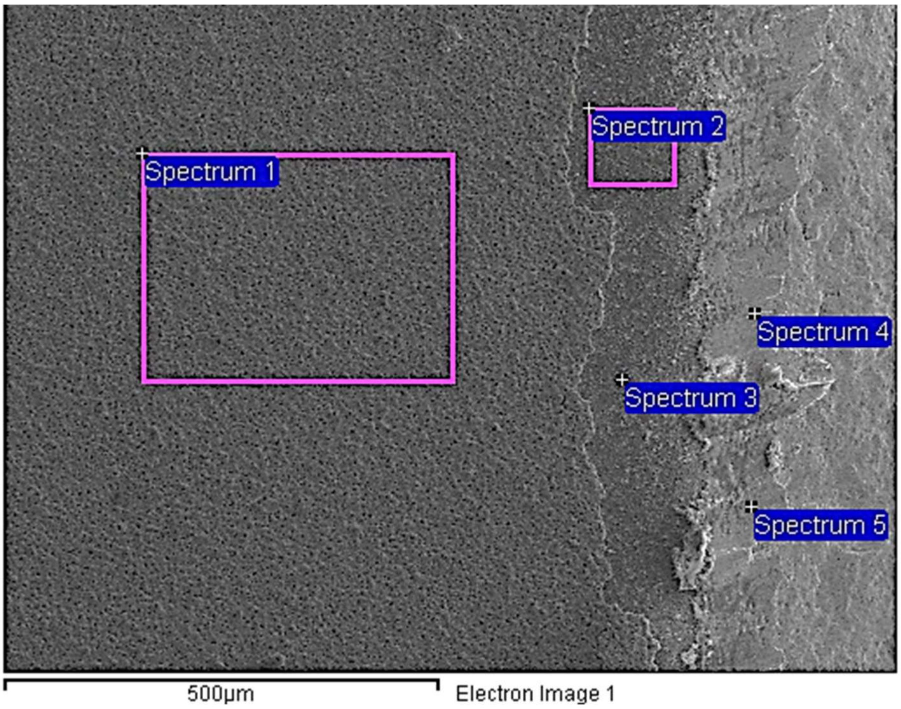

Spectrum	In stats.	B	C	O	Na	Ti	Ni	W
Spectrum 1	Yes	8.06	12.31	59.36	0.47	1.90	7.32	10.58
Spectrum 2	Yes	0.00	3.76	66.32	0.32	26.15	0.27	3.18
Spectrum 3	Yes	2.37	6.96	66.40	0.20	17.08	0.52	6.46
Spectrum 4	Yes	0.00	10.19	15.66		73.73	0.00	0.42
Spectrum 5	Yes	0.00	31.82	33.00		34.82	0.07	0.28
Max.		8.06	31.82	66.40	0.47	73.73	7.32	10.58
Min.		0.00	3.76	15.66	0.20	1.90	0.00	0.28

Figure S2. An example of determining the elemental composition of BWNi sample on the outer and inner surface areas. Spectrum 1 refers to the outer layer, while spectra 2 and 3 characterize the delamination areas (the surface of the inner layer).

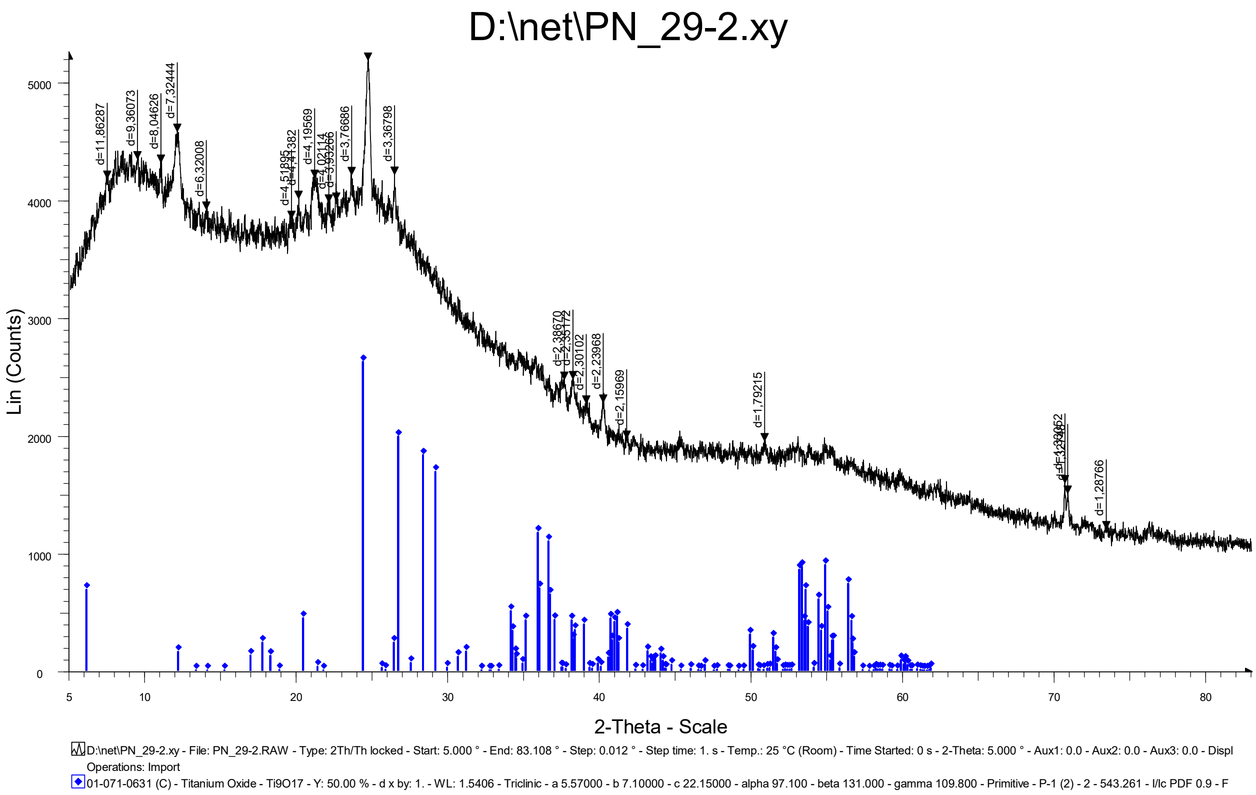

Figure S3. XRD pattern of PWNi sample

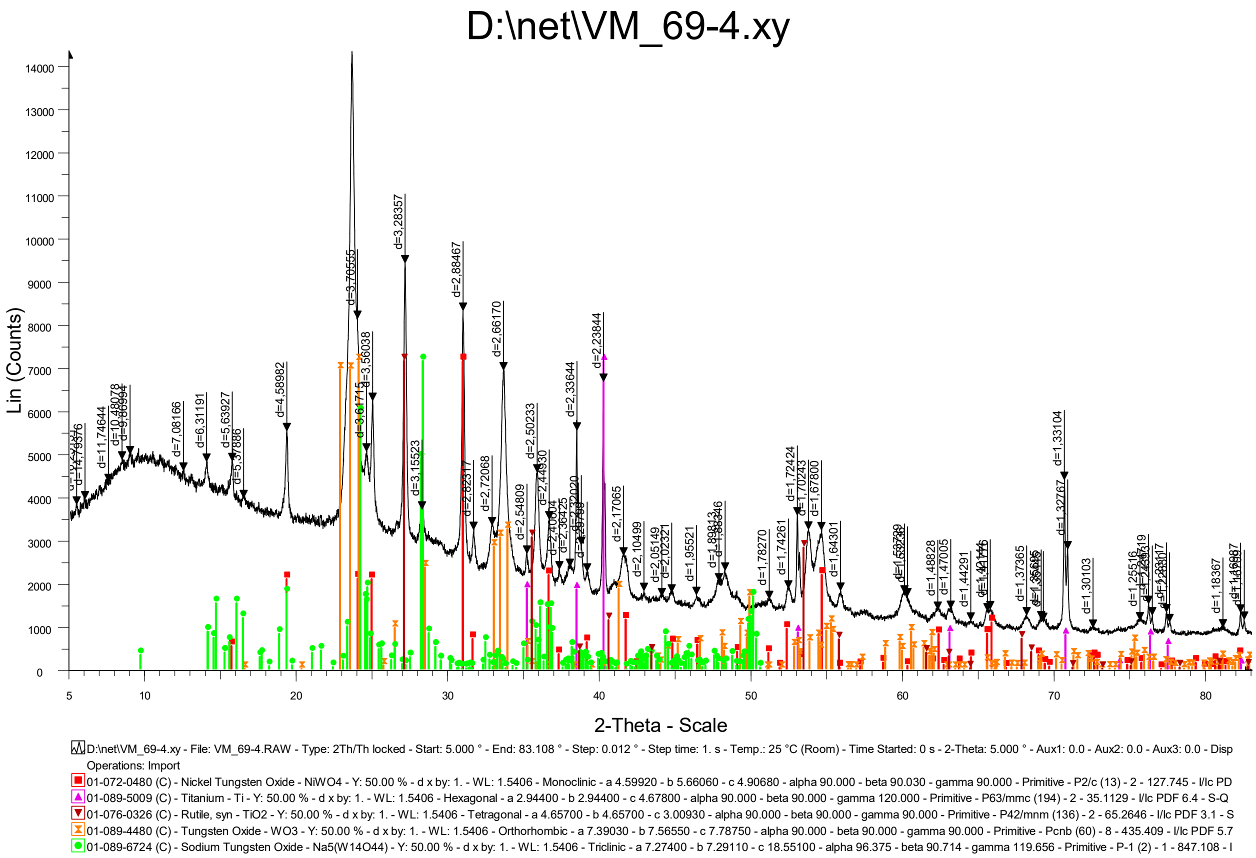

Figure S4. XRD pattern of BWNi sample

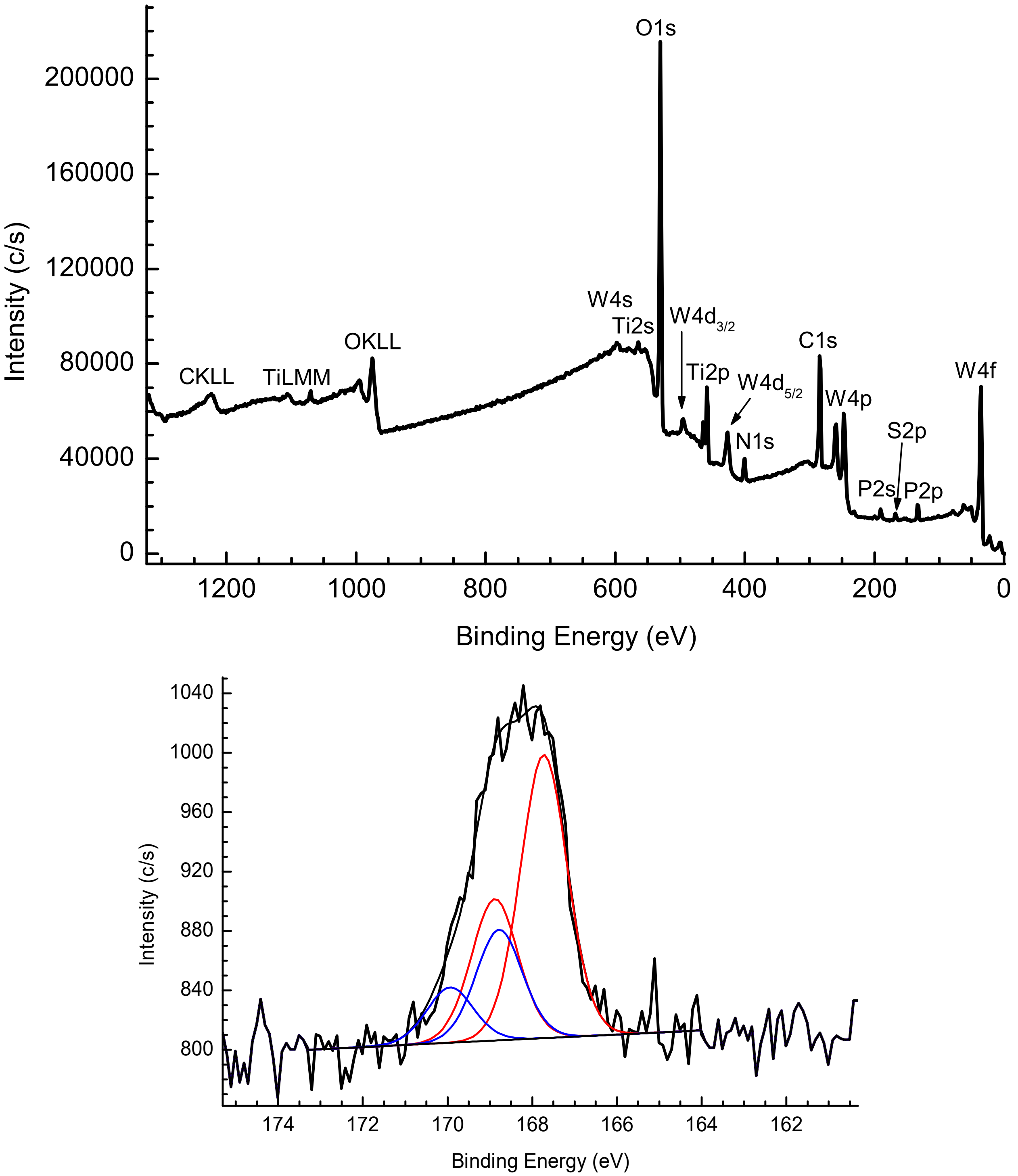

Figure S5. Survey and regional S2p XPS spectra of sample PW after the thiophene oxidation reaction

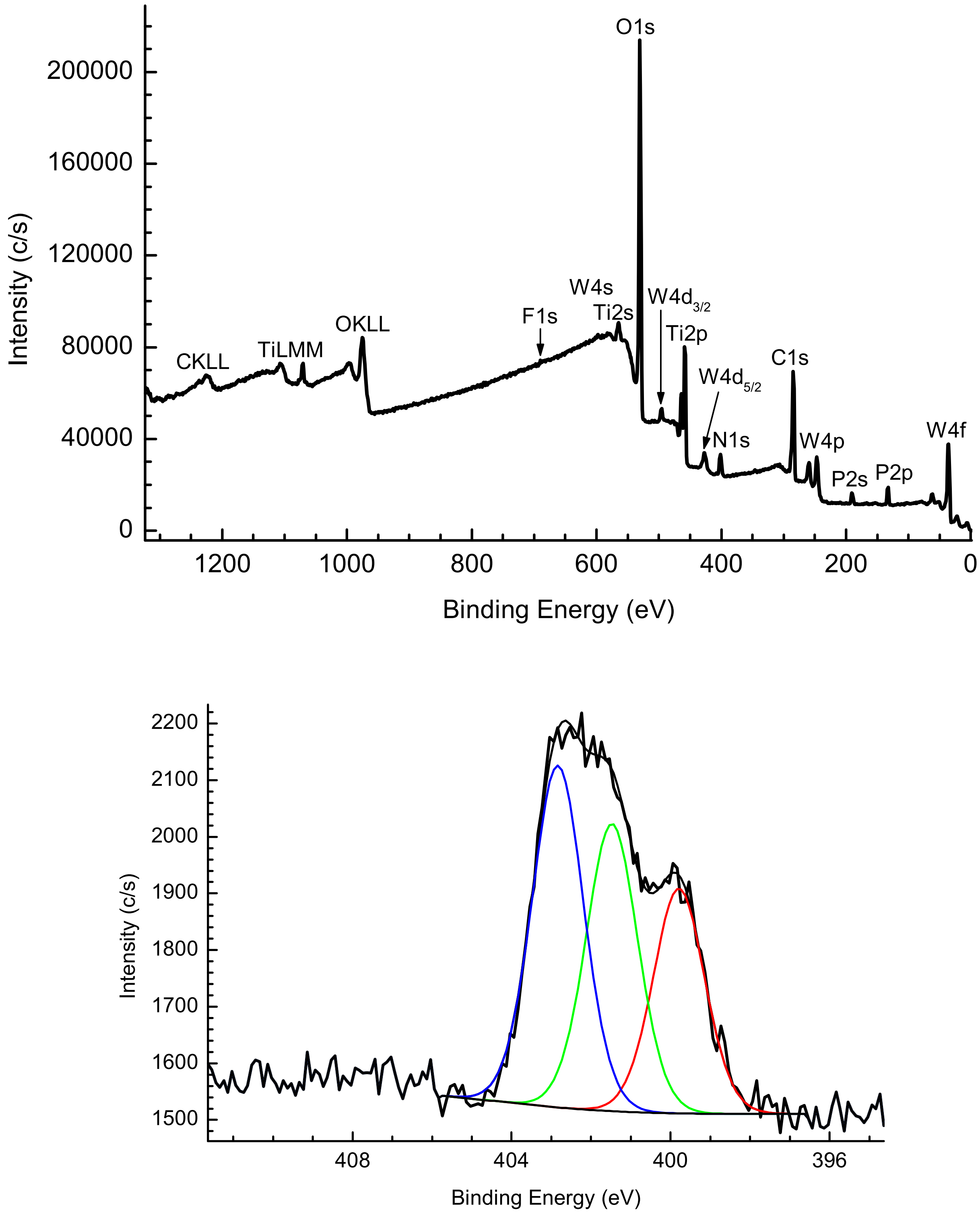

Figure S6. Survey and regional N1s XPS spectra of sample PW after the oxidation of the thiophene - pyridine mixture

Table S1. Binding energies E_b and element concentrations C for surface and subsurface layers of PEO composites

PW

**Sample ▼**	**Line►**	**C 1s**				**O 1s**		**W $4f_{7/2}$**		**P 2p**	**Na 1s**	**N 1s**		**Ti $2p_{3/2}$**		**Total**
PW initial	**C_{total} [at. %]**	**34.4**				**44.7**		**1.0**		**3.4**	**2.6**	**3.6**		**10.2**		**99.9**
	E_b [eV]		287.0	285.0		532.4	530.6	36.0		134.4	1071.8	400		458.8	458.1	
	C [at. %]		13.0	21.4		8.5	36.2	1.0						8.6	1.6	
	C [relative %]		38	62		19	81	100						84	16	
PW after etching	**C_{total} [at. %]**	**7.2**				**60.9**		**3.9**		**5.9**	**3.6**	**3.2**		**15.3**		**100**
	E_b [eV]	288.5		285.0	281.5	532.1	530.6	36.1	33.1	133.6	1071.8	399.8	397.2	459.0	457.8	
	C [at. %]	1.3		4.7	1.2	14.6	46.3	2.7	1.2	5.4		0.9	2.3	13.2	2.1	
	C [relative %]	18		66	16	24	76	70	30			28	72	86	14	
Proposed compounds, groups		OCO	COC	CC, CH	CW, CTi	PO_x, CO_x	WO_x, MO_x	W^{6+} (WO_3, MWO_4)	W^{4+} (WO_2)	P^{5+}	Na^+	N-N, N-H	N-Ti	Ti^{4+} (TiO_2)	Ti-N	

PWFe

Sample ▼	**Line►**	**C 1s**			**O 1s**		**W $4f_{7/2}$**		**P 2p**	**Na 1s**	**N 1s**		**Ti $2p_{3/2}$**		**Fe $2p_{3/2}$**		**Total**
PWFe initial	**C_{total} [at. %]**	**26.6**			**49.4**		**3.8**		**8.7**	**2.7**	**3.7**		**1.7**		**3.3**		**99.9**
	E_b [eV]	287.0	285.0		533.0	531.3	36.0		134.0	1071.6	400		459.4		713.2	711.8	
	C [at. %]	8.5	18.1		8.0	41.4	3.8						1.7		1.6	1.7	
	C [relative %]	32	68		16	84	100						100	0	48	52	
PWFe after etching	**C_{total} [at. %]**	**2.5**			**57.6**		**13.3**		**10.5**	**0.7**	**4.2**		**4.8**		**6.6**		**100.2**
	E_b [eV]	287.0	285.0	283.6	532.7	531.4	36.1	33.4	134.0	1072.0	400.2	397.7	459.5	458.1		710.7	
	C [at. %]	0.8	1.1	0.6	9.6	48.0	10.6	2.7			1.2	3.0	3.6	1.2		6.6	
	C [relative %]	32	45	23	16	84	80	20			28	72	75	25		100	
Proposed compounds, groups		COC	CC, CH	CW, CTi	PO_x, CO_x	WO_x, MO_x	W^{6+} (WO_3, $FeWO_4$)	W^{4+} (WO_2)	P^{5+}	Na^+	N-H	N-Ti	Ti^{4+} (TiO_2)	Ti-N	Fe^{3+}	Fe^{2+}	

PWCo

Sample ▼	Line ►	C 1s				O 1s		W $4f_{7/2}$		P 2p	Na 1s	N 1s		Ti $2p_{3/2}$		Co $2p_{3/2}$		Total
PWCo initial	**C_{total} [at. %]**	**41**				**40.6**		**3.7**		**4.4**	**1.8**	**3.9**		**1.0**		**3.6**		**100**
	E_b [eV]	288.1	286.2	284.8		532.8	531.0	34.7			1071.0	400.8	399.4	458.8		781.6		
	C [at. %]	4.5	11.6	24.7		13.0	27.6	3.7				1.0	2.9	1.0		3.6		
	C [relative %]	11	28	61		32	68	100				26	74	100		100		
PWCo after etching	**C_{total} [at. %]**	**7.8**				**52.4**		**12.9**		**6.7**	**4.6**	**2.2**		**3.9**		**9.5**		**100**
	E_b [eV]		287.0	284.9	283.6	532.6	531.5	35.7	33.8	133.5	1072.8	399.7	397.4	459.2	457.9	782.0	778.5	
	C [at. %]		1.7	5.1	1.0	15.2	37.2	9.0	3.9	6.7		1.0	1.2	3.4	0.5	7.6	1.9	
	C [relative %]		22	66	12	29	71	87	13	91		49	51	87	13			
Proposed compounds, groups		OCO	COC	CC, CH	CW, CTi	PO_x, CO_x	WO_x, MO_x	W^{6+} (WO_3, MWO_4)	W^{4+} (WO_2)	P^{5+}	Na^+	N-H	N-Ti	Ti^{4+} (TiO_2)	Ti-N	Co^{2+}	Co^0	

PWNi

**Sample ▼**	**Line ►**	**C 1s**		**O 1s**		**W 4f$_{7/2}$**		**P 2p**	**Na 1s**	**N 1s**		**Ti 2p$_{3/2}$**		**Ni 2p$_{3/2}$**		**Total**
PWNi initial	**C_{total} [at. %]**	**18.3**		**61.5**		**9.5**		**3.0**	**1.5**	**1.5**		**3.5**		**1.4**		**100.2**
	E_b [eV]	286.7	285.0	532.0	530.6	37.3		134.8	1072.0	400.0		459.1	458.1	854.7		
	C [at. %]	4.4	13.9	17.0	44.5	9.5						0.5	3.0	1.4		
	C [relative %]	24	76	28	72	100						13	87	100		
PWNi after etching	**C_{total} [at. %]**	**2.6***		**60.6**		**16.8**		**6.7**	**1.2**	**3.7**		**5.3**		**3.1**		
	E_b [eV]			532.0	530.8	37.1	34.4	134.8	1072.0	399.5	397.4	458.9	457.7	854.5		
	C [at. %]			9.7	50.9	13.3	3.5			0.7	3.0	4.2	1.1	3.2		
	C [relative %]			16	84	79	21			20	80	80	20	100		
Proposed compounds, groups		COC	CC, CH	PO_x, CO_x	WO_x, MO_x	W^{6+} (WO_3, MWO_4)	W^{4+} (WO_2)	P^{5+}	Na^+	N-H	N-Ti	Ti^{4+} (TiO_2)	Ti-N	Ni^{2+}	Ni^0	

(*) Due to the low concentration, the carbon peak cannot be resolved into its components.

BWNi

**Sample ▼**	**Line ►**	**C 1s**				**O 1s**		**W 4f$_{7/2}$**		**B 1s**	**Na 1s**	**N 1s**		**Ti 2p$_{3/2}$**		**Ni 2p**		**Total**
BWNi initial	**C_{total} [at. %]**	**34.0**				**42.0**		**9.0**		**3.0**	**0.5**	**3.5**		**1.9**		**6.1**		**100**
	E_b [eV]	288.6	286.1	285.0		532.2	530.6	35.6	34.5	190.0	1072.0	399.7	396.9	458.8	457.8	856.1		
	C [at. %]	3.7	8.3	22,0		11.0	31.0	7.0	2.0			3.0	0.5	1.6	0.3	6.1		
	C [relative %]	11	24	65		26	74	78	22			88	12	82	18	100		
BWNi after etching	**C_{total} [at. %]**	**19.4**				**49.0**		**13.0**		**3.5**	**0.6**	**3.8**		**2.0**		**8.6**		**99.9**
	E_b [eV]	289.0	287.0	285.0	283.4	531.9	530.9	35.4	34.0	191.0	1072.0	400.0	397.0	459.2	458.1	856.5	853.2	
	C [at. %]	1.7	1.9	15,0	0.8	12.7	36.3	11.0	2.0			2.9	0.9	1.7	0.3	6.6	2.0	
	C [relative %]	9	10	77	4	26	74	84	16			77	23	87	13	77	23	
Proposed compounds, groups		OCO	COC	CC, CH	CW, CTi	PO_x, CO_x	WO_x, MO_x	W^{6+} (WO_3, $NiWO_4$)	W^{4+} (WO_2)	B^{3+}	Na^+	N-H	N-Ti	Ti^{4+} (TiO_2)	Ti-N	Ni^{2+}	Ni^0	
